# Micro- and Nanoplastics and Fetal Health: Challenges in Assessment and Evidence from Epidemiological Studies

**DOI:** 10.3390/toxics13050388

**Published:** 2025-05-12

**Authors:** Ankica Sekovanić, Tatjana Orct, Zorana Kljaković-Gašpić

**Affiliations:** Division of Occupational and Environmental Health, Institute for Medical Research and Occupational Health, 10000 Zagreb, Croatia; asekovanic@imi.hr (A.S.); torct@imi.hr (T.O.)

**Keywords:** microplastics, nanoplastics, prenatal exposure, birth outcomes, digestion and extraction, pollution, particles

## Abstract

The usage of plastics in life and industrial applications has led to global environmental pollution by micro- and nanoplastics (MPs/NPs). Despite their widespread occurrence in the environment, little is known about their presence in humans and the potential implications for human health, particularly maternal and fetal health during the prenatal and neonatal periods. Studies on experimental animals indicate that exposure to MPs/NPs can lead to neurological abnormalities in offspring and hemodynamic alterations in the placenta and fetal cerebral arteries. These findings underscore the need for further epidemiological studies that examine the effects of MPs/NPs on fetal health during pregnancy, a critical period for neurological development. This review summarizes the existing knowledge on the effects of prenatal exposure to MPs/NPs on fetal development and birth outcomes in humans and provides a detailed overview of the challenges encountered in contamination prevention, quality assurance and quality control in analytical procedures. It also discusses the sampling and digestion methods used for the extraction of MPs/NPs from biological samples of maternal and fetal origin, highlighting the difficulties associated with accurately quantifying these particles in complex biological matrices, identifying the gaps in current research, and suggesting recommendations to improve methodologies for assessing the risks associated with prenatal MP/NP exposure.

## 1. Introduction

Microplastics (MPs), defined as particles ranging from 1 µm to 5 mm in diameter, and nanoplastics (NPs), which are particles smaller than 1 µm in diameter, are synthetic solid particles composed of polymeric materials. These particles can vary in shape, exhibiting either regular or irregular forms, depending on their origin and manufacturing processes [1,2]. Various industries, including packaging, construction, automotive, textile, electrical and electronics industries, household applications, and health and safety equipment, rely on MPs/NPs due to the numerous advantages these plastic materials offer, providing convenience, safety, and enhanced quality of life. However, the release of plastic particles into the environment and food supply has raised significant global concerns, as they become a new form of ubiquitous anthropogenic pollutant [3,4]. While plastic production in Europe has slightly decreased from 62.3 million tonnes in 2018 to 54.0 million tonnes in 2023, largely due to the EU’s Circular Economy Action Plan for plastics, global production continues to rise, from 370.7 million tonnes in 2018 to 413.8 million tonnes in 2024 [5,6]. In Europe, 79.4% of plastics production is fossil-based, while 19.0% is derived from mechanically recycled plastics (13.2% post-consumer and 5.8% pre-consumer recycled). Smaller proportions come from bio-based (1.4%) and chemically recycled (0.2%) sources [6]. Generally speaking, MPs/NPs found in the environment can be categorized into two types: primary and secondary particles. Primary MP/NP polymers are specifically manufactured to be of micro- or nanoscale size for use in various industrial and consumer applications, including air blasting media, cosmetics, and as drug vectors in medicine. Secondary MP/NP particles are formed from the decomposition of larger plastic debris under the influence of physical, chemical, and environmental factors, resulting in the creation of smaller MP/NP particles that further contribute to environmental pollution [7,8,9].

Particles of MPs/NPs are ubiquitous, found in environments ranging from deep-sea sediments [10] to Mount Everest [11]. They have been detected in various environmental compartments [12,13,14], drinking water and food [15,16,17,18,19], and even baby bottles [20]. The primary concern regarding the potential adverse effects of MPs/NPs lies in their role as carriers of a variety of chemicals, including plasticizers and organic and inorganic pollutants [21], which have the potential to disrupt hormones or be chemically toxic [9,22,23]. Endocrine-disrupting chemicals are compounds that affect the endocrine system’s homeostasis by mimicking natural hormones, counteracting their effects, altering their synthesis and metabolism, or modulating the expression of particular receptors [24]. Humans are primarily exposed to MPs/NPs through ingestion and inhalation [25]. Ongoing research is focused on understanding the extent of human exposure via food, drink, and air, as well as the associated health consequences [26]. The adverse impacts of MP/NP exposure can be classified into physical and chemical effects. Physical effects are related to the particle size, shape, and concentration of MP/NP particles adsorbed onto membranes, which can alter cellular functioning [27,28]. Chemical effects stem from hazardous substances embedded in MPs/NPs during their manufacture, including additives such as clay, silica, glass, chalk, some metals, and flame retardants, which are often introduced to improve the properties of polymeric products [29,30]. Furthermore, the surface of MPs/NPs can adsorb biological proteins or biomolecules that enable them to cross the blood–brain barrier, causing disturbances in oxidative and inflammatory balance, changes in neurotransmitter function, and the activity of enzymes crucial for nerve conduction [31,32]. The absorption of MPs/NPs in the body is influenced by several parameters, including the particle’s hydrophobicity, surface charge, functionalization, size, and surrounding protein corona, which may facilitate their entry into the bloodstream after cellular uptake or paracellular diffusion [33]. It has been reported that particles smaller than 150 µm can cross the gastrointestinal epithelium in mammals, while those smaller than 20 µm are capable of passing through cellular membranes and the blood–brain barrier [19,21,34]. With regard to the respiratory system, NP particles and smaller MP particles can penetrate deeply into the lungs, remaining on the alveolar surface and potentially causing lung injury.

The complexity of MP/NP stressors presents significant challenges for analyzing and understanding their impacts, which is attributable to their diverse physical-chemical properties. Despite the widespread presence of MP/NP particles in the environment, knowledge regarding their effects on human health, particularly fetal health, remains limited. Most of what is understood about the effects of MPs/NPs on fetal development is derived from cellular-level studies and/or animal models [35]. Studies conducted on experimental rats exposed to MPs/NPs have shown that these particles can accumulate in the placenta, causing hemodynamic changes in both the placenta and the fetal cerebral artery [36,37], as well as metabolic disorders [38], and alterations in the central nervous system of offspring [39]. Although the precise mechanisms of MPs/NPs transport through the placenta have not yet been clarified, four potential pathways have been proposed: passive diffusion, facilitated diffusion, active transport, and pinocytosis [40]. An ex vivo human placental perfusion model has shown that the placenta absorbs polystyrene (PS) beads [41], which can pass through the placental barrier without compromising its viability [42]. After MP/NP particles enter the body, they interact with target cells. These interactions are influenced by numerous factors, including the particles’ quantity and size, chemical surface properties, and charge of the biological element that interacts with the particles. These interactions lead to the formation of “protein coronas” around the particles, altering their properties and facilitating their translocation [33,43]. According to Yee et al., phagocytosis, macropinocytosis, clathrin-mediated, and caveolae-mediated endocytosis are the pathways of cellular absorption of plastic particles [33]. In studies conducted on pregnant rats orally exposed to polystyrene NP/MPs, these particles were detected in fetal tissues, including the liver, kidney, lung, heart, and brain, in the form of elliptical clusters with irregular borders. This suggests that the particles were transported by fetal tissue’s macrophages [36], which play essential role in tissue remodeling, repair, and angiogenesis. Disruptions in macrophage function may contribute to several neonatal and perinatal conditions, including necrotizing enterocolitis, bronchopulmonary dysplasia, and hypoxic–ischemic encephalopathy [36,44]. Recent studies conducted on cultured neural stem cells (NSCs) and mice exposed to polystyrene NP particles during developmental stages have reported significant abnormalities in fetal brain development accompanied by neurophysiological and cognitive deficits [39]. Additionally, transcriptome and 16S rRNA sequencing studies on mice exposed to MPs during gestation and lactation evaluated the potential effects of maternal MP exposure on F1 and F2 generations [45]. These studies reported alterations in physiological markers associated with glycolipid metabolism in the liver and serum of mothers and their F1 and F2 offspring [45]. These findings underscore the importance of further investigation into the effects of MPs/NPs on fetal health, particularly during pregnancy, which is a crucial period for the development of the fetal neurological system [29,46].

This gap in knowledge motivated us to compile an overview that addresses (1) the current challenges in contamination prevention and the implementation of non-plastic protocols in epidemiological studies, (2) the quality assurance and quality control issues in MPs/NPs determination, (3) the sampling and digestion methods/protocols for ‘extracting’ MPs/NPs from maternal and fetal biological samples, and (4) an extensive critical review of the current data on MPs/NPs presence in maternal and fetal compartments, identifying major research gaps in this field.

## 2. Literature Search Strategy and Selection Criteria

The search strategy of major scientific databases (Web of Science—WoS, PubMed, Science Direct, and Scopus) was based on the combination of the following keywords: *microplastic*, *nanoplastic*, *fetal* or *fetus*, *umbilical*, *placenta*, *maternal*, *breastmilk*, and *amniotic fluid*. To ensure comprehensive coverage, a systematic approach was applied across all databases. The primary keywords (*microplastic*, *nanoplastic*) remained constant, while the third keyword was systematically varied among the additional terms listed above. This approach allowed for a more detailed and targeted retrieval of relevant studies. No time restrictions were imposed, ensuring that all available literature, regardless of publication date, was included in the search. The inclusion criteria were as follows: human subjects, data on the presence of MPs/NPs in samples of maternal and fetal origin (e.g., maternal blood, placenta, cord blood, meconium, amniotic fluid, breastmilk), and environmental exposure of the general population to MPs/NPs through inhalation or ingestion. Only studies that reported original concentration data and were published in English have been considered. The exclusion criteria included review papers, guidelines, letters to the editor, meta-analysis, studies published in languages other than English, studies focused on occupational exposure, and studies containing duplicate data. A total of 215 results matched the query, including 64 review articles, 8 meeting abstracts, and 4 editorial materials, all of which were excluded from consideration. The remaining articles (n = 139) were carefully reviewed, leading to the exclusion of 74 studies involving experimental animals (mice/rats) or cell cultures, 41 studies dealing with environmental organisms or samples, 2 studies investigating baby food, and 5 studies focusing on human populations other than mother and/or fetal/newborn subjects. The final selection comprised 19 research articles: 17 from the WoS database, and 2 from PubMed and Science Direct databases. The earliest articles on this topic were published in 2021 by Ragusa et al. [47] and Braun et al. [48]. Most of the studies were conducted in China (n = 8), followed by Italy (n = 3), the USA (n = 2), and one study each from Thailand, Indonesia, Canada, the Czech Republic, Iran, and Germany. The number of participants/samples in the studies ranged from 2 to 62. Ten studies investigated the association between lifestyle habits or general characteristics of participants and the presence of MPs/NPs in biological samples. Three studies explored the relationship between different maternal/fetal compartments, while four studies examined MP/NP presence in different regions of the placenta. Only three studies investigated the association between MP/NP abundance and birth outcomes, two of which used placenta samples and one of which used amniotic fluid for MP/NP detection.

## 3. Challenges in MP/NP Detection

The identification and quantification of MPs/NPs in human biological samples can be very challenging due to the widespread presence of plastics in the environment, and personal/occupational surroundings. Therefore, meticulous precautionary measures must be implemented at every stage of the analytical workflow to minimize the risk of contamination and ensure the reliability of results. A critical step in MP/NP analysis involves the removal of the organic matrix, which is essential for isolating plastic particles from complex biological substrates. Additionally, a filtration step is required to extract MPs/NPs before instrumental detection. However, these procedures must be carefully optimized to prevent contamination and sample loss while maintaining the accuracy of the analysis. To improve reproducibility and facilitate meaningful comparisons across studies, it is essential to establish standardized protocols for the sampling, processing, and analysis of MPs/NPs in biological samples. These protocols will enable the accurate identification, measurement, and characterization of MPs/NPs, which are among the most challenging analytes to detect in both environmental and biological matrices. The following sections will summarize the current state of knowledge regarding the challenges encountered during the analysis of MP/NP particles in human-derived samples, particularly those of maternal–fetal origin. These challenges encompass issues related to inevitable external contamination, the implementation of quality assurance and quality control protocols, and the processes involved in sampling and digesting biological samples to enable accurate analysis of MP/NP particles.

### 3.1. Preventing Contamination—Plastic-Free Protocol

As mentioned previously, plastic particles are ubiquitous in the environment, making sample contamination during collection, preparation, and analysis almost inevitable. This high risk of contamination complicates analyses of MP/NP presence, requiring researchers to exercise extra care and effort when handling samples to be able to differentiate between MP/NP particles that are actually present in the sample and those introduced through external contamination. In general, to minimize cross-contamination during MP/NP analysis in human biological samples, several key practices should be followed: using non-plastic equipment; avoiding the use of synthetic textiles during sampling and sample handling; implementing rigorous cleaning procedures for working surfaces, tools and consumables; utilizing both procedural and environmental blanks to control for contamination; keeping samples and equipment covered with non-plastic items; and conducting sample handling in clean rooms and/or laminar-flow hoods with controlled air circulation [49].

The majority of the papers in this overview claim that procedural blanks and plastic-free protocols were used during all experimental stages (collection, storage, sample preparation, and analysis). Plastic-free protocols involve using glass or steel tools instead of plastic ones, wearing cotton laboratory coats and latex gloves, and thoroughly cleaning tools and instruments (scissors, tweezers, vials, etc.) with dishwashing liquid followed by rinsing with 70% ethanol and filtered water. Additionally, all liquids used in the experiment, such as ethanol and water (deionized or ultrapure), were filtered through µm-sized filter membranes. Furthermore, authors of the studies that assessed MPs/NPs in samples of maternal and fetal origin have reported that obstetricians and midwives typically wear cotton gloves over inner rubber gloves during delivery. They also ensure that the beds in the delivery room are covered only with cotton towels (which is impractical and can ruin the beds), and avoid using plastic bags for measuring postpartum blood loss, which is common practice in birthing units. While these plastic-free procedures may be feasible in studies with small sample sizes, they become increasingly impractical in large-scale epidemiological studies, particularly in maternity hospitals where dozens of births occur daily. In such settings, where the priority is to ensure the safety and well-being of both the medical staff and the patients, these strict protocols are often unfeasible. The need to provide timely and effective care to a large number of women and protect the health of both mothers and newborns takes precedence over the implementation of stringent plastic-free measures. This makes it nearly impossible to adhere to the meticulous protocols described, as they could disrupt the flow of delivery, which must always focus on safeguarding the health of both mother and child. Based on our prior experience conducting epidemiological research on this vulnerable population [50,51,52], a more comprehensive approach would be to examine every item used in the delivery room, including gloves, mats, and containers, and to take samples of the indoor air in the delivery room for comparison with the actual samples. That approach was also implemented in a study by Braun et al. [48], who analyzed MPs in the airborne fallout and all plastic-containing materials used in laboratory and surgical rooms to evaluate contamination risks. Except for the placenta, these authors also analyzed MPs in meconium, though they did not establish a strict protocol for handling this type of biological sample due to the high risk of environmental contamination, as the sample of meconium, spontaneously emptied from the bowel, was collected in the operating room [48]. In another recent study, Garcia et al. [53] reported that although they took every precaution to minimize contact with plastic materials, the samples of placenta analyzed in their study were originally stored in plastic tubes and were also in contact with plastic tubes during ultracentrifugation. Despite these potential sources of contamination, the authors argued that the contamination levels were consistent across all samples, as each sample was handled in the same manner. Furthermore, they suggested that any contamination from plastic tubes was very low, as most individual polymer concentrations were below the detection limit [53].

A recently published study by Noonan et al. [54] emphasized the critical role of using blank controls to ensure the reliability of the data, showing that samples prepared in a fume hood exhibited higher levels of plastic contamination compared to those prepared on the laboratory bench or inside the laminar flow hood. Only seven of the articles reviewed in this paper incorporated environmental blanks to evaluate contamination from external sources. Ragusa et al. [47,55] reported using environmental and/or procedural blanks in their studies. Environmental laboratory blank samples were collected daily by placing a filter membrane soaked with filtered deionized water in an uncovered Petri dish on a flat surface, while procedural blanks were prepared alongside every batch of samples, undergoing the same processing steps but without the addition of the actual sample [47,55]. In their study of breastmilk, the procedural blanks were found to be free of MP contamination, whereas only nylon fibers were detected in the environmental blanks. The authors concluded that blank correction was not necessary, as the size of the fibers found in blanks (571–3000 µm) was too large to be transferred into the milk through potential transport mechanisms. This was further confirmed by the absence of fibers in analyzed milk samples [55]. Furthermore, Zurub et al. [56] used a plastic-reduced approach, replacing plastic materials with non-plastic alternatives whenever feasible. In cases where this was not possible due to operational procedures and standards of care, items were rinsed with water, and the rinses were analyzed for MPs/NPs. Among the different plastic-containing items used in the delivery room that could not be replaced, only one item—the surgical drape—showed evidence of shedding, with only polypropylene (PP) particles detected in the rinse [56]. Halfar et al. [57] used passive sampling of indoor air into empty Petri dishes lined with filters to monitor contamination from the laboratory environment. Using Fourier Transform Infrared (FTIR) spectroscopy for instrumental MP analysis, they identified only seven particles—six fibers and one fragment—across six blank samples, deciding to exclude the fibers from further analysis, as they are prone to airborne dispersion. Using a different detection method, Weingrill et al. [58] reported that procedural blanks were free of MP contamination, while certain environmental blanks had fibers on the filter’s surface. The authors excluded surface fibers and focused solely on particles within the glass filters, which is achievable with Raman spectroscopy, rendering the normalization of the blanks redundant. Hasanah et al. [59], in their study of MPs/NPs in the feces of pregnant women using FTIR microspectroscopy, also controlled for environmental contamination by passive collection of work area fallout in Petri dishes filled with deionized water, but unlike the previously mentioned studies, they did not detect any MPs/NPs in the blank samples [59]. The review of the aforementioned studies underscores the necessity for careful planning and execution and detailed reporting of sampling and blank-testing procedures. Regardless of the detection technique employed for identifying MP/NP particles in different samples, ensuring the accuracy and reliability of results necessitates a systematic approach to both environmental and procedural blank tests. Environmental blanks are crucial for identifying contamination from the surrounding environment, whereas procedural blanks evaluate contamination introduced during sample handling, preparation, or analysis. The execution of these tests facilitates the detection of undesirable external factors, thereby ensuring that the data accurately represent solely the intended microplastic contamination. The correction of analytical results based on blank-test outcomes is essential to eliminate false positives or skewed data that may compromise the integrity of the study. A standardized approach to blank testing is essential for achieving accurate, reliable, and reproducible results in microplastic research, thereby enhancing the robustness of the conclusions derived from these studies.

### 3.2. Quality Assurance and Quality Control

Quality assurance and quality control (QA/QC) of measurement data are essential to ensure the accuracy and reliability of findings, as the accuracy and uncertainty of measurement data are fundamental for the comparison of results between different studies. However, the lack of standardization in sampling techniques significantly hampers the ability to make valid cross-study comparisons. Prata et al. [60] also critically addressed this issue, though their discussion was limited to the analysis of MP/NP particles in water and sediment samples [60]. Furthermore, the development and implementation of quality control measures and procedures are significant challenges in MP/NP research, particularly in ensuring the integrity of data throughout the sample collection and analysis process. Sample contamination is a particular concern in this field, as MPs/NPs are omnipresent in the environment, with the potential for contamination occurring at multiple stages (during sampling, digestion, and analysis). While the majority of the studies outlined essential quality control procedures to prevent cross-contamination, further optimization is still required, particularly in providing more detailed explanations of sampling and pretreatment procedures. Many additional factors can also influence the likelihood of contamination, such as the time required for MP/NP extraction from the sample and the sample analysis, as well as the number of procedural steps involved. Many studies have addressed QA/QC concerns by adopting plastic-free protocols and relying primarily on procedural blanks to detect and account for potential contamination. However, as previously mentioned, only a limited number of studies incorporate environmental blanks, which are just as, if not more, important than procedural blanks. Environmental blanks are essential given the ubiquitous presence of MP/NP particles, which can easily contaminate samples during sampling, digestion, and analysis. By employing both procedural and environmental blanks, researchers can gain a clearer understanding of how samples are affected during preparation, helping to neutralize the influence of cross-contamination and ensuring its potential impact on the results. In a recent study, Prata et al. provided recommendations for reducing and controlling air and cross-contamination during sampling, processing, and identification of MP/NP particles [49]. These procedures include handling samples in a room with controlled ventilation and limited access for laboratory personnel, performing weekly cleaning, using a laminar flow hood, covering solutions with aluminum foil, utilizing glass and metal materials, washing materials with acid and filtered water, and filtering working solutions. Most importantly, they emphasize the need for both procedural and environmental blanks [49]. However, many studies fail to provide a detailed description of how the environmental blanks were conducted. Some studies fail to report the results obtained from blanks, neglect to describe how these results were handled, or omit reporting how they were considered when processing the results (whether they were deducted from the samples or not).

Although a very important element of QA/QC, the recovery of the methods for the ‘extraction’ and detection of MPs/NPs was addressed and reported in only three studies. Two of these, conducted by Liu et al. [61,62], used soil samples to evaluate recovery rates, as the authors noted that it is extremely difficult to obtain biological tissue samples free of plastics [61,62]. The third study, conducted by Zhu et al. [63], focused on assessing recovery rates for the three most common plastic polymers (polyethylene (PE), polystyrene (PS), and polyvinyl chloride (PVC)) [63]. However, none of these studies provided a detailed explanation of their methods, such as whether they used commercially available reference materials, which specific reference materials were used, whether real samples were spiked with polymer particles, whether alternative reference samples (e.g., in-house produced materials of defined composition and particle size) were employed to evaluate recovery rates, or what size the particles present in the reference material were. The limited number of studies addressing the recovery likely reflects the scarcity of appropriate commercially available standard reference materials—those that provide both qualitative and quantitative data, include information on particle size distribution, and mimic the irregularly shaped plastic particles typically found in environmental settings—for method validation and recovery assessment. These materials have only recently become accessible, and their high cost further hinders their widespread implementation, posing an additional barrier to standardization in current MP/NP research.

In conclusion, future studies on MP/NP particles should include the use of matrix spikes to accurately quantify recovery rates for specific detection techniques. For example, representative standard reference materials containing well-defined types, sizes, and quantities of microplastic particles could be used to spike samples, which would then undergo the same pretreatment and analytical procedures as real samples. Moreover, it is crucial that recovery rates are clearly defined and reported, with acceptable recovery rates typically set at 80% or higher, consistent with other analytical techniques, as this threshold ensures reliable and reproducible results. For complex or challenging matrices, lower recoveries may be deemed acceptable if they are adequately accounted for and justified. Incorporating matrix spikes and establishing clear recovery criteria can enhance the accuracy, consistency, and comparability of MP/NP studies, thereby improving the reliability of findings and advancing the field.

### 3.3. Sampling and Digestion Methods

Over the past decade, several comprehensive reviews have been published that focus, either in part or in entirety, on methods for the detection, identification, and quantification of MP/NP particles in various environmental and biological matrices [64,65,66,67,68]. This paper aims to narrow the scope of the existing literature by highlighting the methodologies related to sampling and digestion techniques employed in the analysis of MPs/NPs in human biological samples, particularly those of maternal and fetal origins.

Defining the sampling procedure for biological samples with precision and clarity is essential to ensure the reproducibility and reliability of results. However, many studies included in this review provide only brief or incomplete descriptions of the sampling methods employed for placenta collection, which can hinder the consistency and comparability of findings across studies. The placenta is the most commonly utilized sample for assessing maternal and fetal exposure to MPs/NPs in studies that focus on sensitive populations, such as mother–newborn pairs (Table 1). In fact, the presence of particles in the placenta was examined in 63% (12 out of 19) of the studies reviewed. This organ plays a critical role as a functional interface between the mother and fetus, facilitating the exchange of oxygen and essential nutrients while also removing carbon dioxide and metabolic waste. As such, the placenta serves as an invaluable biological sample, not only as an early indicator of fetal exposure but also as a reflection of maternal exposure to environmental pollutants. However, the placenta presents a considerable challenge for analysis due to its complex structure, comprising two distinct components: the fetal component, known as the chorionic plate, and a maternal component, the decidua. Furthermore, acquiring the placenta in a hospital setting for research purposes can be quite demanding, especially in MP/NP studies, where contamination control is paramount. The process of subsequent sampling of the placental tissue in the laboratory presents additional challenges, as discussed in the previous section on potential sources of MPs/NPs contamination and the importance of plastic-free protocols. Given these challenges, it is essential to implement standardized protocols for both the sampling of the representative placental tissue and the digestion of those samples, ensuring consistency and reliability across studies. However, a significant number of the studies encompassed in this overview either provided brief or incomplete descriptions of how the placenta was handled in the delivery room [58,69] or failed to report this information at all [47,53,56,57,63,70,71]. Only a limited number of studies offered detailed and comprehensive descriptions of their sampling methods. In an effort to prevent external contamination during placenta handling, one pilot study [48] described a rigorous placenta sampling technique. Following a cesarean section, the placenta was delivered by gentle traction of the umbilical cord and held above a metal kidney dish while the cord was cut with metal scissors to avoid direct contact. Liu et al. [61,62] reported that, in order to prevent the contamination of the placenta during delivery, sterile cotton gloves were utilized by obstetricians and midwives to help women at childbirth [61,62]. Similarly, Ragusa et al. [72] reported that cesarean sections were conducted by operators wearing cotton gloves and without inserting their hands into the uterus. During vaginal births, midwives wore cotton gloves in the final stages of fetal expulsion to minimize the risk of placenta contamination [72]. While these measures of avoiding contact with the placenta aim to prevent contamination, they are impractical in real-world clinical settings where medical personnel, under the guidance of an obstetrician, routinely inspect each placenta to make sure that no fragments remain in the uterus—a critical procedure to prevent life-threatening complications for the mother [73]. Additionally, as mentioned before, the use of cotton gloves in these situations may not be optimal, as they do not provide an adequate barrier against bloodborne diseases and other potential contaminants.

An additional challenge in placenta sampling for the MP/NP analysis is the selection of a representative sample. Given the placenta’s dual composition, consisting of both fetal and maternal components, strict sampling protocols are crucial to ensure accurate and consistent results. However, many of the studies included in this review provided only brief summaries of their sampling procedures, often lacking sufficient detail for full reproducibility. For instance, Garcia et al. [53] reported that placental samples were collected in cuboidal sections, with each section circumferentially excised at a distance of 4 cm from the umbilical cord insertion site. The maternal decidua and fetal chorion-amnion were carefully excised and discarded to minimize the risk of potential contamination [53]. Braun et al. [48] described sampling a 1 × 1 × 1 cm section of the placenta, which was washed several times with ultra-pure water or, alternatively, the outer surface of the placenta was removed, and only the core placental tissue was sampled, which aligns with the methodology described by Garcia et al. [53]. In contrast, Ragusa et al. [47] collected samples from the maternal side, fetal side, and chorioamniotic immediately after birth, but did not provide any additional details on the sampling technique. In a separate study, Ragusa et al. [72] reported the collection of three 5 mm placental samples taken from the intraparenchymal portion, again without further procedural details [72]. Amereh et al. [69] described the collection of three portions from different areas of the placenta—one from the chorioamniotic membrane and one from both the maternal and fetal sides —and stored these samples in bottles without any further treatment [69]. Other studies also varied in their sampling methods. Liu et al. [61,62] and Zhu et al. [63] sampled the fetal side of the placenta and broke it into small pieces, while Halfar et al. [57] and Yun et al. [70] targeted the central portion of the placental basal plate. However, these studies did not elaborate on the handling or preparation of samples before digestion. Weingrill et al. [58] described a different approach, where two placental cotyledons were carefully excised using steel scalpels and washed in phosphate-buffer saline (PBS) solution to minimize background plastic contamination [58]. A recent study by Zurub et al. [56] provided a more detailed description of the sampling procedure, specifying that they collected micro-dissections of the placenta from the chorionic plate (fetal side), chorionic villous tissue (maternal-fetal interface), and basal plate (maternal side), cutting between the placental margin and the umbilical cord insertion [56]. All these varying approaches to placental sampling make it nearly impossible to compare results across studies. Since each method targets different regions of the placenta, and given the heterogeneity of placental tissues, these sections likely contain different concentrations of microplastics. As a result, these differences in sampling techniques can introduce substantial inconsistency, further complicating the interpretation of results and hindering cross-study comparisons.

After the placenta, the meconium, breastmilk, and amniotic fluid are the most frequently used biological samples for evaluating prenatal exposure (Table 1). Meconium, which forms in utero around the 13th week of pregnancy and accumulates in the fetal colon until birth, is considered a reliable indicator of fetal exposure to harmful substances [74]. Meconium sampling is much simpler than placenta sampling, with studies generally collecting it under sterile conditions using fecal collectors. However, the timing of meconium collection varies across studies. Braun et al. [48] reported sampling meconium directly in the operating room immediately after delivery, while Li et al. [75] collected meconium by scraping the upper portion from cloth diapers. They did not specify the exact timing of collection, although they reported concurrent collection of the blank samples from the clean areas of the diapers to account for contamination [75]. In contrast, Liu et al. [61,62] and Zhu et al. [63] also collected the upper portion of the meconium within the first 24 h after delivery, but without collecting blank samples [61,62,63].

Another important sample for assessing newborn exposure is breastmilk. Breastmilk provides the required antibodies, energy, and nutrients during the first few months of life [76]. Therefore, the assessment of harmful substances in breastmilk is of paramount importance, as mothers are exposed to various harmful substances from the environment that can transfer into the milk and adversely affect infant health [77]. For breastmilk sampling, providing a detailed methodology is essential, as the timing and method of collection can influence the results. Ragusa et al. [55] provided a detailed description of their breastmilk collection procedure. In accordance with World Health Organization guidelines, samples were collected one week postpartum to minimize any discomfort or harm to the breast tissue. Briefly, their method involved forming a C-shape with the thumb and forefinger and gently squeezing toward the ribcage to release milk [55]. The only drawback of this description was the absence of a detailed protocol for cleaning the breast prior to sampling, a step recommended by Liu et al. [61] and Saraluck et al. [78], although those studies lacked a detailed description of the milk collection process.

The amniotic fluid is the first environment in which the fetus grows and develops, serving as the barrier against infections and providing essential growth factors that support the normal development and growth of fetal organs and tissues [79]. Despite its importance in fetal development, amniotic fluid has been used as a biological sample to assess fetal exposure to MP/NP particles in only three studies [57,80,81]. This limited use is likely due to the invasive nature of amniocentesis, the procedure used to obtain amniotic fluid, which requires careful procedures to maintain the sterile conditions of the amniotic fluid and prevent infection. In the studies mentioned, samples were collected in a similar manner, by using sterilized surgical steel needles and borosilicate glass syringes. The only difference between these studies lies in the timing of sample collection: Halfar et al. [57] collected samples during the amniocentesis, while the other two studies obtained samples during cesarean section deliveries [80,81].

**Table 1:** Sampling and digestion methods used for ‘extracting’ MPs/NPs from human biological samples of maternal and fetal origin.

Type of Sample	Sampling	Mass/Volume of Samples	Digestion Method	Method of Detection	Source
placenta	cuboidal sections 4 cm from the cord insertion; maternal decidua and fetal chorion-amnion were cut off	0.4 g	Samples were digested in glass vials by adding 10% KOH (3× the tissue volume) and incubating at 40 °C for 72 h with continuous agitation. The supernatant was transferred to ultracentrifuge tubes, 200 μL of 100% EtOH was added, and the mixture centrifugated at 100,000× *g* for 4 h. The dark brown, transparent supernatant was removed and pellets washed three times with absolute ethanol. Samples were dried for 24 h at room temperature and stored in glass vials.	fluorescence microscopy, FTIR, Py-GC-MS	[53]
placenta	micro-dissections sampled from the basal plate (maternal surface), chorionic villous tissue (maternal-fetal region), and chorionic plate (fetal surface)	1 g	Samples were digested in Erlenmeyer flasks by adding 100 mL of 30% H_2_O_2_, then incubated at 55 °C for 5 days at 100 rpm. After 5 days, 50 mL of 30% H_2_O_2_ was added and incubation continued for additional 6 days. The digest was vacuum filtered through 2.7 μm glass fiber membrane, followed by rinsing with 10 mL of De-H_2_O (at 55 °C). Membranes were placed in Petri dishes and dried in a hood.	Raman microspectroscopy	[56]
placenta	central portion of the base	2 g	In a glass bottle, 4 mL of KOH solution (15%, *v*/*v*) was added to the sample, and incubated for 48 h in a thermostatic shaker at 40 °C. The supernatant was then filtered through 0.8 cm^2^ sterile glass filter, and the filter was stored in a container.	Raman microspectroscopy, Py-GC-MS	[70]
placenta	two cotyledons were cut into smaller fragments and washed in filtered (1.6 μm) PBS	50 g	In glass bottles, 10% KOH solution (1:8, *w*/*v*) was added to the samples and incubated at room temperature during 7 days. The digestates were filtered through 1.6 μm glass fiber filter membranes, which were dried at ambient temperature.	light microscopy and Raman spectroscopy	[58]
placenta	portion of placenta—maternal and fetal side	1 g	A 10% KOH solution was added to the glass beakers containing samples at 1:30 (*w*/*v*) ratio and incubated for 3 days at 50 °C while agitated at 120 rpm. The obtained solutions were filtered through 10 μm stainless-steel membranes. Membranes were then placed in Erlenmeyer flasks, 240 mL of KHCO_2_ solution (1.50 g/cm^3^) was added, and the content was ultrasonicated for 30 min. Obtained suspension was filtered through another 10 μm stainless-steel membrane, and absolute EtOH was used to reduce sample volume to 0.5 mL.	LDIR	[71]
placenta	portions of maternal and fetal side, and chorioamniotic membrane	not specified	In a glass container, a 10% KOH solution (1:8 *w*/*v*) was added to the samples and incubated for 7 days at room temperature. The digests were than filtered through 1.6 μm filter paper, which were dried at ambient temperature before storage in Petri dishes.	light microscopy and Raman microspectroscopy	[69]
placenta	portions of maternal and fetal side, and chorioamniotic membranes	23.3 ± 5.7 g	A 10% KOH solution was added to glass containers containing samples (1:8 *w*/*v*) and incubated for 7 days at ambient temperature. The digestates were filtered via 1.6 μm filter membranes, dried at ambient temperature, and stashed in Petri dishes.	light microscopy and Raman microspectroscopy	[47]
breastmilk	manually milked into a glass vials	10–15 g	A 10% KOH solution was added to the samples, and incubated for 48 h at 40 °C. The digestates were filtered via 1.6 μm filter membranes, placed in Petri dishes and allowed to dry at ambient temperature.	FT-Raman spectroscopy	[78]
breastmilk	manual milked into a glass container	4.16 ± 1.73	A 10% KOH solution was added to glass flasks with samples (1:10 *w*/*v*) and incubated for 48 h at 40 °C. The digestates were filtered via 1.6 μm filter membranes, dried at ambient temperature and stashed in Petri dishes.	Raman microspectroscopy	[55]
amniotic fluid	aspiring by a glass syringe and a 20-gauge surgical steel needle	not specified	The weighed samples were digested in glass beakers with concentrated nitric acid (HNO_3_, 68%) for 3 h at 95 °C. The resulting suspension was filtered via 13 μm stainless-steel membrane, rinsed several times with Milli-Q water and anhydrous EtOH, and ultrasonicated in absolute EtOH at 40 kHz for 40 min. The membranes were rinsed again with absolute EtOH; the EtOH was reduced to 200 μL, transferred to reflective glass plate, and dried at ambient temperature before analysis.	LDIR	[81]
meconium	scraping the top portion of meconium from cloth diapers by sterile fecal collectors	0.4–4.7 g	Samples were freeze-dried, transferred to glass tubes, and crushed with glass rods. A mixture of petroleum ether and alcohol (4:1, *v*/*v*) was added, content sonicated, and samples were left to stand until they separated into layers. The supernatant was discarded, and fresh solution was added before sonication. This process was repeated until a colorless solution was obtained. The colourless supernatant was discarded, and substrates were dried under a nitrogen gas flow. Next, 5 mL of 65% HNO_3_ per gram of meconium was added, and left overnight in a cold water bath. The dissolved substrates were digested at 80 °C for 4 h. If the solution remained muddy, an additional 2 mL of HNO_3_ was added, and digestion continued for additional 30 min. to obtain transparent solution. To ensure complete digestion, 5 mL of 30% H_2_O_2_ was added, and the mixture was incubated at 80 °C for 30 min. The solution was filtered via 10 μm stainless-steel membrane, rinsed several times with water at 70 °C, and the membranes were placed into Petri dishes and dried at ambient temperature or at 50 °C in a drying oven.	ultra-depth three-dimensional microscope and micro-FTIR	[75]
maternal stool	morning stool	25 mg	Feces was mixed with 12.5 mL 1% phenol and 62.5 mL distilled water and vortexed. The mixture was placed in a Petri dish, covered with aluminum foil, and incubated at 60 °C for 48 h. Dry samples were crushed and transferred to 200 mL glass bottles where dry feces was dissolved with 10% KOH (ratio 1:3) during 2 weeks until they fused and changed from solid to colloidal.	stereomicroscope and FTIR	[59]
cord blood	using sterile disposable syringe	0.1–1.0 g	Placenta and meconium samples were freeze-dried before digestion. In glass beakers containing samples, 10 mL of 30% H_2_O_2_ was added, and incubated for 3 h at 70 °C. Then, 2 mL of conc. HNO_3_ was added, and incubated for additional 2 h. The digestate was filtered via a 1 μm glass fiber filters.	Raman microspectroscopy	[63]
placenta	fetal side close to the umbilical cord				
meconium	top portion scraped by wooden cotton swabs from the surface of the diaper				
maternal and cord blood, amniotic fluid	using syringe	5 mL	The samples were digested in glass beakers by adding conc. HNO_3_ (3 times the sample mass). The mixture was digested at ambient temperature for 48 h. If the samples were not fully digested, an additional amount of HNO_3_ was added, and digested for aditional 24 h. The digestion was completed by concentrating the sample on a heating plate at 60 °C to approximately 1 g. The samples were then vacuum filtered through 13 μm stainless steel filter membranes, which were washed several times with Milli-Q water and EtOH. The membranes were ultrasonicated in EtOH, evaporated to 150 μL, solution quantitatively transferred to a reflective glass slide, and left to dry at ambient temperature for further analysis.	LDIR	[80]
umbilical cord, fetal membrane	cut with metal scissors	3 g			
placenta	maternal surface near cord insertion; umbilical cord and fetal membrane were cut off				
amniotic fluid	aspiring by surgical steel needle (20-gauge) and borosilicate glass syringe	2.5–7 mL	A 30% KOH solution was added to glass tubes containing amniotic fluid (ratio undefined) and digested at ambient temperature for 24 h. The mixture was filtered through 1 μm glass fiber filters, and filter membranes left to dry in Petri dishes for one week.	FTIR	[57]
placenta	central part of basal plate	0.5 g	Samples were treated with KOH solution (10% *v*/*v*) at 37 °C for 2 h, and at ambient temperature for 22 h. After digestion, solutions were filtered via 1 μm glass fiber filters, stashed in Petri dishes and dried in a exiccator for one week.		
placenta	taken from fetal side and sectioned in portions	not specified	In a glass beaker, conc. HNO_3_ (68%) was added to the sample, left for 48 h, and heated for 3 h at 95 °C. The mixture was filtered through a 13 μm stainless-steel filter, rinsed with Milli-Q water and absolute EtOH, and the filter ultrasonicated in absolute EtOH for 30 min. The filter was rinsed again several times with absolute ethanol, and the obtained solution filtered via additional 13 μm stainless-steel filter. The ultrasonication step was repeated, followed by additional rinsing with absolute EtOH. The final filtrate was evaporated to 200 μL, and transferred to a reflective glass slide.	LDIR	[61,62]
meconium, infant feces	top portion by spatula from the surface of the diaper				
breastmilk	manual milked into a glass container				
placenta	blocks (1 × 1 × 1 cm) of whole placental tissue and “core” placental tissue	not specified	Samples were sieved through a 50 μm stainless steel and rinsed into a beaker with 30% H_2_O_2_. The mixtures were digested for about 5 weeks (meconium) or 7 weeks (placenta) with multiple additions of H_2_O_2_ to eliminate organic matter. Residuals were sieved through 50 μm stainless steel filters and placed in 0.05 M NaOH. Digestates were transported onto a 50 μm sieve (stainless-steel), and rinsed with Milli-Q water into a glass container. The solutions were filtered via 0.2 μm membrane filters, which were stashed in Petri dishes, and dried at 60 °C overnight.	FTIR	[48]
meconium	spontaneously emptied from the bowel and transferred into glass bottles	not specified			

Other biological matrices linked to mother–newborn pairs, such as maternal and cord blood [63,80], maternal stool [59], infant membrane [80], and feces [61], have also been used to assess exposure to MP/NP particles, although these matrices have been utilized much less frequently. Unfortunately, none of these studies provided a detailed description of their sampling protocols and preparation procedures, nor did they offer sufficient detail on the components of their quality assurance and quality control measures. For example, although it was mentioned that fecal samples were collected by scraping from diapers [61], and that blood samples were initially obtained using disposable plastic syringes and then transferred into glass tubes containing anticoagulant [63], these studies either did not include sampling blanks or provided only vague descriptions of the preparation and analysis of blanks, which are crucial for identifying potential contamination during the collection process. Additionally, there was no mention of recovery calculations, which are critical for assessing the accuracy of both sampling and analytical methods. This lack of methodological transparency and standardization complicates the comparison of results across different studies. As a result, it is currently challenging to assess the full impact of MP/NP exposure on the health of both mothers and fetuses when relying on these biological matrices.

Following the initial sampling of biological materials, additional challenges in MP/NP analysis arise during the digestion of the biological matrix, aimed at removing interfering organic matter, and the ‘extraction’ of MP/NP particles from the samples. These steps are crucial for enabling the subsequent detection and characterization of the particles. All the methods currently applied for the digestion and ‘extraction’ of MP/NP particles from human biological samples of maternal and fetal origin are summarized in Table 1. The first stage in the detection of MPs/NPs in biological samples is digestion. A variety of digestion reagents—ranging from alkaline and acidic to oxidizing agents—have been employed in this stage, but the findings across studies remain inconsistent. The duration of digestion varies significantly depending on the protocol used, with some of the procedures taking several months, which may result in loss or secondary contamination that could influence the detection of MPs/NPs. Among the different methodological approaches for the digestion of placenta samples, the majority of the studies (67%, or 8 out of 12) used potassium hydroxide (KOH) to digest the biological matrix, typically employing a 10% solution [47,53,57,58,69,71,78], while only one study utilized a 15% KOH solution for this purpose [70]. The incubation conditions (time and temperature) varied across studies, with some researchers incubating the samples at room temperature for seven days [47,58,69], while others used 40 °C for 48 h [70,78] or 37 °C for two hours, followed by 22 h at room temperature [57]. After incubation, the samples were generally filtered through filters with defined pore sizes, most commonly 1.6 µm, although one study employed a 1 µm pore size [57]. This choice of pore size depends on the technique used for the MP/NP analysis, as the smallest detectable particle diameter varies by method. More precisely, filters with pore diameters smaller than the minimum detectable particle size are used as filtration media, which ensures that all particles of interest are retained on the filter’s surface. A slightly different digestion method for placenta samples was applied by Garcia et al. [53] and Zhu et al. [71]. Garcia et al. [53] reported that after incubating placenta samples with 10% KOH at 40 °C for 72 h, isolated pellets were washed with 100% ethanol and dried at room temperature for 24 h. This procedure allows for the extraction of plastic particles from the tissue without plastic degradation [53]. Zhu et al. [71] used 10% KOH solution and digested the samples at 50 °C for three days, followed by additional treatment with potassium formate solution in an ultrasonic bath for 30 min [71]. In addition to KOH, other reagents, such as HNO_3_ [61,62,80] and H_2_O_2_ [48,56], either used alone [48,56,61,62,80] or in combination [63], have also been employed for placenta digestion. Prolonged processes of separating plastic particles from non-plastic compounds were described by Braun et al. [48] and Zurub et al. [56].

These processes could extend from several days to even a few weeks, often requiring additional doses of H_2_O_2_ to completely digest the organic material. Both Sun et al. [80] and Liu et al. [61,62] used concentrated HNO_3_ for 48 h to eliminate the organic components in placenta samples, but applied different temperatures (combination of room temperature and 60 °C *vs*. combination of room temperature and 95 °C for three hours). Concentrated HNO_3_ [61,62] and a combination of HNO_3_ and H_2_O_2_ [63,75] have also been employed for the digestion of meconium samples. Li et al. [75] examined three commonly used pretreatment methods for the digestion of human and animal feces and meconium, such as H_2_O_2_, HNO_3_, and a combination of Fenton’s Reagent and HNO_3_, and reported incomplete digestion of meconium with these methods. As an alternative, they developed a novel approach that involved lyophilizing meconium, extracting a colorless supernatant using a petroleum ether-ethanol mixture (4:1, *v*/*v*), and then digesting the substrate with HNO_3_ (65%) and H_2_O_2_ (30%), first at room temperature and then at 80 °C [75]. However, there is increasing evidence that prolonged exposure to strong reagents, such as HNO_3_ and H_2_O_2_, particularly under elevated temperatures, may increase the risk of external contamination, cause plastic fragmentation, discoloration, and MP/NP loss, lead to lower recovery rates or the overestimation of MP/NP concentrations, or result in false-positive findings [82,83]. To improve the accuracy and reliability of MP/NP analysis, it is essential to conduct further studies aimed at standardizing the procedures for digestion and ‘extraction’ of MP/NP particles from various human biological samples. Future research should thoroughly evaluate all existing digestion methods and systematically test a range of conditions, such as reagent volumes, incubation temperatures, and durations, to identify the most effective protocols. Additionally, it is crucial that these tests are performed on identical types of biological samples to ensure consistency in the results. Focusing on the harmonization of digestion and extraction techniques across studies will enable researchers to compare findings more effectively, improve the precision of particle detection, and establish more reliable methods for assessing MP/NP exposure. Standardization will enable the establishment of quality control measures that guarantee reproducibility across various laboratories and research environments, which is essential for enhancing our understanding of the health effects of MP/NP particles.

## 4. Review of the Currently Available Data—Impact on Fetal Health

A thorough search of the literature was conducted for this review, with Table 2 summarizing the published results about the occurrence of MP/NP particles in human biological samples from a sensitive population group, namely mother–newborn pairs. The screening and detection of MP/NP particles has been published in 21% of articles (4 out of 19 reviewed). Among these, two studies used archived placenta samples [53,58], while the remaining two used placenta and/or meconium to develop a digestion method [75] and protocol for MP/NP detection [48]. Four studies examined the differences in MP/NP particles distribution and abundance across different regions of the placenta. One of these studies found no significant variation in the number of particles between different areas of the placenta (e.g., basal plate, chorionic villous, and chorionic plate) [56], while another study observed that 33% of the particles were found on the fetal side, 49% on the maternal side, and 18% in the chorioamniotic membranes [69]. Ragusa et al. [47] examined only six placenta samples and detected 12 MP fragments across four samples (fetal side—5 particles, maternal side—4 particles, chorioamniotic membranes—3 particles), with sizes ranging from 5 to 10 µm. In a separate study, Ragusa and colleagues utilized scanning electron microscopy (SEM) and transmission electron microscopy (TEM) to demonstrate for the first time that MP/NP compatible fragments were detected and localized in different intra and extracellular compartments of human placenta, linking these fragments with ultrastructural alterations in certain intracytoplasmic organelles (endoplasmic reticulum and mitochondria) that had not been observed in healthy pregnant women [72]. These findings suggest that MPs/NPs may accumulate in the placenta, potentially weakening and crossing the placental barrier, thereby threatening fetal development. The transfer of endogenous and exogenous substances, including MPs, across the placenta is greatly influenced by several factors, including the surface area of the chorionic villi (approximately 14 m^2^), the structure of the fetal barrier, and fetal perfusion from both the maternal and fetal sides. Given the short lifespan of the placenta, typically around 8 months, which is brief when compared to an individual’s entire lifespan, the accumulation of MP/NP particles within this limited timeframe is particularly concerning and may have significant implications.

It has been well recognized that lifestyle habits have a significant impact on the body’s absorption of MP/NP particles, and 11 out of the 19 reviewed studies have examined these behaviors and their relationship to MP/NP findings in studied biological samples. A recent study on the placentas of 50 Chinese women found microplastic particles in 31 samples, with the most common polymer types being acrylonitrile butadiene styrene (ABS), polytetrafluoroethylene (PTFE), and PS [70]. The authors also investigated the association between MPs’ size and abundance in the placenta and maternal demographic characteristics, such as age, body height and weight, and BMI, and reported no significant correlation between analyzed parameters [70]. This aligns with the findings of Zhu et al. [71], though their study identified polyvinyl chloride (PVC), PP and polybutylene succinate (PBS) as the dominate polymers in placenta samples [71]. An Italian study of six placenta samples, in which they recorded participants’ food consumption and use of personal care products (toothpaste and cosmetics) in the week leading up to delivery, also reported no significant association between identified MPs and these factors [47]. Similarly, the same group of authors examined MP levels in breastmilk and found no association between detected MPs and maternal data, such as age, use of personal care products, fish/shellfish consumption, consumption of drinks from plastic bottles and of food in plastic packaging [55]. By contrast, several Chinese studies [61,63,80] conducted on the placenta, meconium, infant feces, cord blood, and breastmilk have reported significant associations between lifestyle factors and the accumulation of MP particles. Hence, higher total MP abundance and polyamide (PA) levels were observed in the placentas of women who consumed more than 2 L of water daily compared to those who drank less than 2 L [61]. Frequent tea consumption (>3 times weekly) was also associated with higher MP levels in meconium [63]. Moreover, maternal BMI was associated with increased MP levels in amniotic fluid [80], while women using scrub cleaners or toothpaste more than twice a week had higher levels of PE in their placenta than those who used these products less frequently [61]. Furthermore, higher MP concentrations were found in endometrium of participants who chewed gum, consumed milk tea, and drank carbonated drinks in comparison to those who did not consume these items [84]. The influence of maternal lifestyle habits has also been documented in studies from Thailand, Indonesia, and Iran [59,69,78]. In these populations, higher seafood consumption was associated with higher MP concentrations in maternal stool [59], while consuming takeaway food as opposed to home-cooked meals was linked to higher MP levels in the placenta [69]. Saraluck et al. [78] compared two groups of subjects, one with MPs present in breastmilk and the other without MPs in breastmilk, and found that the latter group had a higher percentage of individuals who washed their hands frequently and a lower incidence of mastitis, breast engorgement, and low breastmilk supply [78]. These findings confirm that lifestyle habits, particularly those related to beverage and product consumption, may play a significant role in the accumulation of MP particles in maternal and fetal biological samples.

Only three studies in the literature have investigated the influence of MP/NP presence on newborn outcomes [69,70,81]. Xue et al. [81], who investigated MP levels in the amniotic fluid of 40 Chinese women, found a positive association between MP levels and both seafood and bottled water intake, and a negative association between MP levels and week of pregnancy and birth weight [81]. A study conducted on 43 pregnant women, which evaluated the relationship between plastic particles in placental tissue and neonatal anthropometric measurements [69], found that, in the intrauterine growth restriction (IUGR) group, MP levels in the placenta were negatively associated with birth outcomes [69]. In contrast, a recent Chinese study did not find any significant differences in maternal and fetal outcomes related to MP/NP exposure [70].

Several studies have concurrently analyzed MP/NP particles in multiple biological samples, including three studies conducted on the Chinese population [62,63,80] and one on the Czech population [57]. One study reported a negative association between MPs in meconium and cord blood, while MPs in cord blood were positively associated with MPs in the placenta [63]. Further analysis of these data suggests that meconium (2.23–77.17 particles/g) contains 1.6–8.4 times more particles than the placenta (1.37–9.15 particles/g) and 2.2–4.9 times more particles than cord blood (0–15.6 particles/g). While these findings may initially suggest increased fetal exposure to MPs, a direct comparison of MP levels in the placenta and cord blood indicates otherwise. Namely, MPs were detected in all placenta samples but in only 55% of analyzed cord blood samples, implying that the higher levels of MPs in meconium may be attributed to environmental contamination, as previously reported by Braun et al. [48]. Another study conducted in China concurrently analyzed MP particles in breastmilk, infant feces, meconium, and placenta [61]. When comparing the published levels, meconium contained approximately three times more MPs (54.1 particles/g) than the placenta (18.0 particles/g), while infant feces had 1.5 times more particles (26.6 particles/g) than the placenta (18.0 particles/g). In contrast, MP levels in breastmilk and placenta were comparable (20.2 and 18.0 particles/g, respectively) [61]. In a separate study, the same authors reported positive correlations between PVC in the placenta and PA in meconium, as well as between PP in the placenta and total MP levels, PA, and PE in meconium [62]. They also examined the microbiota of meconium and placenta, revealing that PS content in meconium is negatively associated with the Chao index of the meconium microbiota, that PE content in the placenta is inversely related to several placental microbiota genera, and that PA and polyurethane (PU) in meconium affect several meconium microbiota genera [62]. In order to explore potential relationships between MP levels in maternal venous blood, fetal appendages, and umbilical cord blood, Sun et al. [80] analyzed MP particles using LDIR spectroscopy. Their findings revealed comparable amounts of MPs in the placenta (4.675 particles/g) and amniotic fluid (4.795 particles/g), whereas cord blood contained approximately half as many particles (2.726 particles/g). A direct comparison of blood samples further revealed that MP levels in cord blood were three times lower (2.726 particles/g) than in maternal blood (8.176 particles/g). Despite these observed differences, correlation matrix analysis found no significant correlations between MP levels in maternal blood, fetal appendages, and umbilical cord blood [80]. A Czech study on ten women with preterm birth was the only European study to examine multiple compartments concurrently [57]. It reported that in seven out of ten subjects, MP levels in amniotic fluid were lower than those in the placenta. The findings from both Halfar et al. [57] and Sun et al. [80] indicate that the placenta may serve as a partial barrier, limiting the transfer of MPs into fetal blood and amniotic fluid. However, further research is needed to elucidate the mechanisms of MP placental transfer and its potential effects on maternal and fetal health.

Based on the available literature, there is a notable lack of research on the relationship between lifestyle habits and MP/NP exposure in the European region, with only one Italian study [55] and one Czech study [57] addressing this topic [53]. Furthermore, other existing studies, most of which were conducted on the Chinese population, report inconsistent findings regarding the effects of MP/NP exposure on maternal and fetal outcomes. Research examining the effects of MP/NP exposure on neonatal parameters also remains scarce. Given that MP/NP particles have been detected in the placenta, amniotic fluid, and breastmilk, concerns about their potential negative impacts on fetal and newborn health are increasing, as they are more susceptible to the toxic effects of these particles than the general population. As a result, further research is urgently needed to clarify the implications of MP/NP exposure for newborn health and development.

## 5. Final Remarks

**Figure 1:**
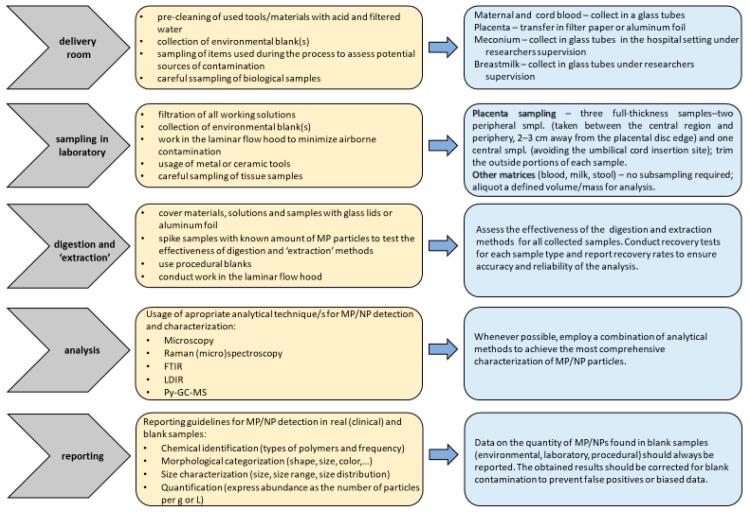
Illustration of recommendations for clinical and laboratory practices in the sampling, digestion, and identification of MPs/NPs in human biological samples.

As highlighted in a recent comprehensive review, MPs/NPs represent a global environmental problem [21] due to their infiltration into the food chain and potential accumulation in organs/tissues of living organisms [7,85]. In vitro studies have shown that the physicochemical characteristics of MPs/NPs, such as their size, shape, chemical composition, and concentration, may have a major impact on their toxicity [86]. However, the mechanisms behind the absorption, distribution, and accumulation of MPs/NPs in various target organs remain unclear. This knowledge gap underscores the need for further research that should be focused on diverse population groups, especially vulnerable individuals, and conducted over a broad geographical range to provide a clearer understanding of exposure levels and their potential adverse effects. Exposure to MPs/NPs through sources like tap water, bottled water, and food packaging may differ significantly depending on local and regional variations in waste management, water treatment processes, and food safety regulations. Therefore, to make meaningful comparisons between studies and generate reliable information that will contribute to the efforts to prevent and reduce plastic pollution and its negative impact on human health, additional efforts are required to establish standardized protocols for MP/NP determination in biological samples. A lack of standardized methodologies was also highlighted in a recent World Health Organization (WHO) report, which addressed exposure to MPs/NPs through diet and inhalation and examined their potential effects on human health [87]. Establishing clear standards for MP/NP analysis is critical for ensuring that study findings are comparable across different research efforts. These standardized methods should include protocols for using blanks (e.g., environmental, laboratory, procedural) to control contamination, as well as washing procedures for laboratory equipment, guidelines for minimizing contamination risks, and guidelines for adjusting results to account for contamination from blank samples. Our general recommendations for clinical and laboratory practices during sampling, digestion, and identification of MPs/NPs in human biological samples are illustrated in Figure 1. Proper documentation on appropriate equipment (e.g., air filtration systems) and procedures for reporting contamination levels must also be incorporated. Another important consideration in the standardization of procedures for reporting contamination with MPs/NPs is the inconsistency in how different types and shapes of microplastics are described. Commonly used terms to characterize MP/NP particles include pellets, pieces, and fibers. However, other forms such as films, ropes, filaments, sponges, foams, rubber, and microbeads are also frequently mentioned in the literature and are significant contributors to plastic pollution. The variety of shapes and forms that microplastics can take complicates efforts to establish a unified classification system. Alongside the need to standardize detection protocols, it is essential to recognize that researchers across different studies may use different terminologies to describe the same types of plastic particles. This lack of uniformity in classification can hinder comparisons and the consolidation of data across research efforts, making it more difficult to draw reliable conclusions. These challenges were highlighted by Frias and Nash in their recent focus study [1], underlining the importance of resolving these inconsistencies to improve the accuracy and comparability of MP/NP research.

Currently, there are only three human studies in the literature that examine the impact of MPs/NPs on prenatal development and fetal health. A significant limitation of these studies is their small sample size, each involving no more than 50 subjects. This limited sample size reduces statistical power, hindering the ability to evaluate the impact of confounding variables on research outcomes and making it difficult to draw reliable conclusions regarding the effects of MPs/NPs on human health, particularly concerning both the mother and fetus. To establish a clear link between MP/NP exposure and health outcomes, it is essential to conduct high-quality observational studies that involve larger sample sizes, and comprehensive sampling across all compartments of the maternal–placental–fetal unit. Maternal exposure to MP/NP particles can be estimated through the analysis of blood, urine, and stool samples. Among these, stool analysis requires the highest level of caution due to its susceptibility to environmental contamination, which could lead to misinterpretation of results. In maternal–newborn epidemiological studies, the placenta serves as a valuable biomarker for dual exposure, encompassing both maternal and fetal factors. However, due to the structural and functional heterogeneity of placental tissue, obtaining representative samples is crucial to ensure accurate and reliable results. To achieve this, we recommend cutting off three full-thickness placental samples: two peripheral samples taken between the central region and the periphery (2–3 cm away from the edge of the placental disk) and one sample from the central region, avoiding the area of umbilical cord insertion. These sections should be treated and analyzed separately to account for potential variations in MP/NP distribution across different regions of the placenta. To minimize the risk of external contamination with MPs/NPs, the outer portions of each sample should be carefully trimmed before processing. MP/NP analysis should then be conducted independently on both the peripheral and central sections to assess potential differences in particle accumulation within the placenta. Fetal exposure to MPs/NPs can also be evaluated using umbilical cord blood, meconium, and amniotic fluid. However, each of these biological matrices presents unique challenges. Meconium, like maternal stool, is highly prone to contamination, and its standardized collection is challenging, as it is often obtained by parents without researcher supervision. Amniotic fluid sampling poses even greater challenges due to the highly invasive nature of the procedure. Additionally, if the amniotic sac ruptures before sampling, collection becomes impossible. These limitations likely explain why amniotic fluid has been examined in only three studies to date [57,80,81]. As a result, umbilical cord blood is the most commonly used sample for evaluating fetal MP/NP exposure. To minimize contamination during blood sample collection, we strongly recommend using glass tubes whenever possible. If glass tubes are not feasible due to study limitations, an alternative approach is to employ a tube blank to help identify and exclude potential external contamination from plastic vacutainers. The blank should contain filtered water and remain in contact with the tube for the same duration as the blood samples. Neonatal exposure can be assessed through analyses of breastmilk and infant feces. However, as previously discussed regarding maternal stool and meconium, fecal sampling for MP/NP analysis presents significant contamination risks. A similar issue exists with breastmilk collection, as it is typically performed by participants rather than researchers, increasing the likelihood of external contamination. This lack of standardized sampling conditions may lead to an inaccurate assessment of neonatal MP/NP exposure during the early stages of development. Therefore, future studies should implement rigorous protocols to ensure reliable and uncontaminated sample collection. In addition, the existing research emphasizes the necessity for further investigation into how MPs/NPs are transferred from mother to fetus, as well as the potential connection between maternal and prenatal exposure to these particles and adverse birth outcomes. Given that plastic particles are both persistent and ubiquitous in the environment, conducting additional studies is crucial to fully understand their effects on fetal health *in utero*. Future research should also investigate the potential long-term developmental consequences of such exposure, which will provide important information for the development of future regulations designed to protect the health of both mothers and their children.

At present, the majority of studies investigating MP/NP exposure in mother–fetus/newborn pairs have been conducted on the Chinese population, while research from other regions remains scarce. This limited representation from other regions may restrict the generalizability of the findings due to geographical biases. Among the most important geographical factors influencing MP exposure are the degree of environmental degradation and the effectiveness of plastic waste management. Countries with high plastic production and consumption rates, such as China and the United States, are more likely to have elevated MP levels in their environments. Additionally, regions with inadequate plastic waste management may experience more widespread contamination of water, food, and air, increasing human exposure. For example, China, responsible for around 33% of global plastic production [6], has encountered enduring challenges in plastic waste disposal, despite recent legislative initiatives aimed at reducing pollution. In contrast, several EU member states have implemented strict plastic consumption and waste disposal rules, which may contribute to reduced MP contamination in the environment and, consequently, in human biological samples. These variations in environmental contamination levels could result in varying MP detection rates across different geographical regions. As dietary consumption is a primary source of MP intake, variations in dietary habits may further contribute to regional variances in MP exposure, with contamination levels varying based on the source and packaging practices. Additionally, populations living in urban and industrialized areas with high levels of air pollution, stemming from vehicle emissions, synthetic textiles, and industrial activities, may be more exposed to airborne MPs than those in rural areas. Government policies and public awareness of plastic pollution may also potentially influence regional MP exposure. Regions with strict plastic regulations, such as the European Union, may have lower human exposure levels, whereas those with less stringent standards may face greater MP exposure. Socioeconomic disparities may also influence MP exposure, as communities with limited access to clean drinking water, fresh food, and effective waste management systems may be more vulnerable to MP exposure due to their reliance on plastic-packaged food and water, processed foods, and inadequate sanitation facilities. In conclusion, geographical bias may have an important influence on the outcomes of MP research in human biological samples due to variations in environmental contamination, air pollution, dietary habits, lifestyle patterns, socioeconomic factors, and legal restrictions. To reduce geographical bias, future research should include multi-regional studies using standardized methodologies; use harmonized protocols for MP sample collection, digestion, and detection; account for regional differences in diet, air quality, and plastic use when interpreting results; and increase research efforts in understudied regions, particularly low-income and developing countries, to provide a more comprehensive understanding of global MP exposure.

## Figures and Tables

**Table 2:** Characteristics, sample types, and main findings of the reviewed studies.

Study Design	Type of Sample	Type of Delivery	n	Country	Detection Method	Size Range (μm)	Types of Debris	Study Findings	Source
pilot observational cohort study	breastmilk	not relevant	59	Thailand	FT-Raman spectroscopy	not specified	PP, PE, PVC, PS, N	MPs were detected in 23 out of 59 breastmilk samples. The authors compared samples with and without detected MPs, revealing that a higher percentage of individuals in the non-detected group washed their hands regularly, washed their hands after feeding, or used washing products designed for babies and mothers. Conversely, the group containing detectable MPs had a higher percentage of individuals experiencing mastitis, breast engorgement, and low breastmilk supply. Also, the bacterial microbiota composition differed between the analyzed goups.	[78]
prospective study	maternal and cord blood, amniotic fluid, umbilical cord, fetal membrane	cesarean section	12	China	LDIR	20–500	ACR, BR, CPE, FKM, PA, PE, PET, PMMA, PP, PS, PU, PVC	Sixteen types of MP materials were detected in umbilical cord (10.397 particles/g), maternal blood (8.176 particles/g), fetal membrane (6.561 particles/g), amniotic fluid (4.795 particles/g), placenta (4.675 particles/g) and umbilical blood (2.726 particles/g). Among all MPs detected in the six sample types, only ACR abundance in maternal blood was higher than in amniotic fluid. Also, total MPs in amniotic fluid was positively correlated with mothers BMI and age.	[80]
not specified	cord blood, placenta, meconium	vaginal/cesarean section	9	China	Raman microspectroscopy	100–400	CEL, PB, PBDT, PEA, PEGMA, PET, PPG, PVA, PVS, PA, PCL, PECH, PE, PI, PNB, PP	MPs were detected in all placenta (total: 34 particles; abundance: 1.37–9.15 particles/g) and meconium samples (total: 80; abundance: 2.23–77.17 particles/g), as well as in 5 out of 9 cord blood samples (total: 14 particles; abundance: 0–15.6 particles/g). Meconium MPs were negatively associated with MPs in cord blood, while MPs in cord blood were positively correlated with MPs in placenta. Additionally, the meconium of individuals who drank tea more than 3 times/week contained lower number of MPs than those who drank tea less than 3 times/week.	[63]
not specified	amniotic fluid	acute cesarean section	40	China	LDIR	20–500	CPE, EVA, PA, PE, PET, PP, PU, PVC, SBS	The average abundance of MPs detected in 32 out of 40 amniotic fluid samples was 2.01 ± 4.19 particles/g. MPs levels were positively associated with seafood and bottled water consumption, and negatively associated with week of pregnancy and birth weight.	[81]
not specified	placenta	not reported	50	China	Raman microspectroscopy	1.03–6.84	PTFE, PS, ABS, PC, PP, PE, PVC	40 MP particles were found in 31 out of 50 placentas, with an average size of 2.35 ± 1.25 µm. No significant association was found between MP size and demographical characteristics (mothers age, BMI, height, and weight, week of pregnancy, newborns’ outcomes, and sex)	[70]
					Py-GC-MS		PTFE, PC		
archived samples	placenta	vaginal	2	USA	fluorescence microscopy and FTIR	>20	PS, PP, PE, PMPS, PET	MPs and NPs were detected in 62 placentas analyzed by Py-GC-MS (abundance: 6.5–685 μg/g tissue). PE and PVC were the most prevalent polymers. The authors concluded that there was no diffusion of MPs from plastic tubes into frozen tissues, as the levels of individual polymers were below the detection limit.	[53]
			62		Py-GC-MS		PE, PVC, N66, SBR, ABS, PET, N6, PMMA, PU, PC, PP, PS		
not specified	maternal stool	pregnant women	30	Indonesia	stereomicroscope and FTIR	200–4900	PET, PA, N, CPE, HDPE, EP	359 MP particles, ranging from 0.2 to 4.9 mm, were found in maternal stool (25 g). Women with moderate to high seafood consumption had higher MP amounts compared to those with low seafood consumption.	[59]
not specified	placenta	vaginal/ elective cesarean section	10	Canada	Raman microspectroscopy	2–60	PE, PP, PS, PVC, PMMA	Plastic (average abundance: 1 ± 1.2/g tissue) and non-plastic particles (average abundance: 4 ± 2.9/g tissue) were found in all samples, ranging in size between 2 and 60 µm. The most frequent polymers were PE, PP, PS and PVC. No differences in MP levels were observed either between the types of delivery or across different placenta regions (basal plate, chorionic villous and chorionic plate).	[56]
not specified	meconium	not reported	16	China	ultra-depth three-dimensional microscope and micro-FTIR	>10	not specified	MPs were not detected in any samples. The authors tested three different digestion methods and developed their own pretreatment procedure.	[75]
archived frozen samples	placenta	cesarean sections	30	USA	light microscopy, and Raman spectroscopy	0–50	PP, PVC, PU, PVA, PET, PE, PA, ABS, PC, PA	Particles were found in 60% placentas from 2006, 90% placentas from 2013, and 100% placentas from 2021. A significant difference in MP size was observed between 2013 (6.24 ± 0.57 μm) and 2021 (5.14 ± 0.75 μm). The number of MPs per 50 g of placenta tissue was higher in the 2021 samples compared to those from 2006 and 2013.	[58]
not specified	placenta	not reported	17	China	LDIR	20.3–307	PA, PAM, PBS, PC, PE, PET, PP, PS, PVC	MP particles were identified in all placentas (average abundance: 2.70 ± 2.65 particles/g; range: 0.28–9.55 particles/g). The majority of MPs (80.29%) were smaller than 100 µm. Detected shapes were: fragments (67.32%), fibers (22.22%), films (9.15%), and subspherical particles (1.31%). No significant relationships were found between MPs abundances, polymer types, sizes, and ages.	[71]
observational cohort study	placenta, amniotic fluid	preterm birth	10	Czech Republic	FTIR	1–500	CPE, PVC, PE, HDPE	The number of detected MPs ranged from 0 to 8 in amniotic fluid, and from 0 to 10 in placenta. Only one patient exhibited a greater number of MPs in the amniotic fluid than in the placenta, whereas 7 patients had more MPs in the placenta than in the amniotic fluid.	[57]
pilot prospective study	placenta, meconium, infant feces, breastmilk	vaginal	18	China	LDIR	20–500	PA, PU, PMMA, PET, PE	MP particle abundance was: placenta: 18.0 particles/g, meconium: 54.1 particles/g, feces: 26.6 particles/g, and breastmilk: 20.2 particles/g. The levels of total MPs and PA in the placenta were higher in women who consumed more than 2 L of water daily than those who consumed less than 2 L. Also, PE levels in the placenta were higher in women who used scrub cleaners or toothpaste > 2 times per week than those who used these products < 2 times per week.	[61]
pilot prospective study	placenta, meconium	vaginal	18	China	LDIR	20–500	PU, PA, PE, PET, PVC, PTFE, PET, POM, EVA, CPE, PS	The median MP particle abundance was 18.0 particles/g in placenta and 54.1 particles/g in meconium. PP in the placenta was positively correlated with total MPs, PA, and PE in meconium, while PVC in the placenta was positively correlated with PA in meconium. Placental EVA and POM were negatively correlated with meconium CPE. PS in meconium was inversely associated with the meconium Chao index of meconium. PE in the placenta was negativelly associated with placenta microbiota genera. Additionally, total MPs, PA, and PU in meconium had impact on some genera of the meconium microbiota.	[62]
pilot observational study	breastmilk	not relevant	34	Italy	Raman microspectroscopy	1–12	NC, PE, PVC, PP, CPE, PVA, PEVA, PMMA, ABS	MPs were found in 76.5% of samples (26 of 34), with abundance ranging from 0.13 to 2.72 particles/g and sizes ranging from 2 to 12 µm. The majority of particles (47%) were in the 4–9 μm range, 29% were smaller than 3 μm, and 24% were larger than 10 μm. No association was observed between MPs and patients’ data (age, use of personal care products, consumption of food in plastic packaging, fish/shellfish consumption, or beverages intake).	[55]
case–control study	placenta	vaginal/cesarean section	43	Iran	light microscopy and Raman microspectroscopy	<50	PE, PS, PET, PP	MPs were identified in all 13 IUGR pregnancies (abundance: 2–38 particles/placenta) and in 4 out of 30 normal pregnancies. Of the detected MPs, highest number was detected on the maternal side (49%), followed by fetal side (33%), and chorioamniotic membrane (18%). MPs abundance was higher in individuals who drank bottled water than those who drank boiled tap water, and in those who ate takeaway food compared to those who consumed home-cooked meals. Furthermore, MPs abundance was inversely associated with birth outcomes in the IUGR group.	[69]
cross-sectional	placenta	vaginal/cesarean sections	10	Italy	scanning electron microscopy and transmission electron microscopy		not specified	MPs were identified in all analyzed samples of placenta. Particles compatible with MPs were identified in different placental compartments (surface of placental villi, inside cells of different placenta cellular layers, and in the extracellular environment).	[72]
pilot observational preclinical study	placenta	vaginal	6	Italy	Light microscopy and Raman microspectroscopy	5 or 10	PP	An amount of 12 MP particles were found in 4 placentas (fetal side—5 fragments, maternal side—4 fragments, chorioamniotic membranes—3 fragments. Among these, 3 were PP, and other 9 were classified as pigments commonly used in paints, coatings, adhesives, plasters, finger paints, polymers and cosmetics. Almost all detected particles were close to 10 μm, except for two particles which were close to 5 μm.	[47]
pilot study	meconiu, placenta	cesarean section	2	Germany	FTIR	>50	PE, PP, PU	A protocol was developed for the detection of MPs > 50 µm in placenta and meconium in real-life clinical setting. Both meconium and placental tissue were positive for PE, PP, PS, and PU. Notably, only PU was detected in airborne fallout from the operating room, indicating a potential source of background environmental contamination.	[48]

ABS—acrylonitrile butadiene styrene; ACR—acrylates; BR—butadiene rubber; CEL—cellulose; CPE—chlorinated polyethylene; EP—ethylene propylene; EVA—ethylene-vinyl acetate; FKM—fluororubber; HDPE—high-density polyethylene; IUGR—intrauterine growth restriction; NC—nitrocellulose; N—Nylon; PAM—polyacrylamide; PA—polyamide; PB—polybuten isotactic; PBDT—polybutadien phenyl terminated; PBS—polybutylene succinate; PC—polycarbonate; PCL—polycaprolactone; PE—polyethylene; PEA—polyethylene adipate; PECH—polyepichlorohydrin; PEGMA—polyethylene glycol ehtylether methacrylate; PET—polyethylene terephthalate; PEVA—poly(ethylene-co-vinyl acetate); PI—polyisoprene hydrogenated; PNB—polynorbornene; PMMA—polymethyl methacrylate; PMPS—polymethylphenylsiloxane; POM—poliacetal; PP—polypropylene; PPG—poly propylene glycol; PS—polystyrene; PTFE—polytetrafluoroethylene; PU—polyurethane; PVA—polyvinyl alcohol; PVC—polyvinyl chloride; PVS—poly vinyl stearate; SBR—styrene-butadiene rubber; SBS—styrene-butadiene-styrene.

## Data Availability

No new data were created or analyzed in this study. Data sharing is not applicable to this article.
